# Counselors’ Clinical Use of Definitive Drug Testing Results in Their Work with Substance-Use Clients: a Qualitative Study

**DOI:** 10.1007/s11469-015-9569-7

**Published:** 2015-07-21

**Authors:** Adam Rzetelny, Barbara Zeller, Nicholas Miller, Kathy Egan City, Kenneth L. Kirsh, Steven D. Passik

**Affiliations:** Millennium Health, 16980 Via Tazon, San Diego, CA 92127 USA; Housing Enterprise for the Less Privileged/Project Samaritan Inc (HELP/PSI), New York, NY USA; Millennium Research Institute, San Diego, CA USA

**Keywords:** Addiction, Substance-use, Treatment, Therapy, Counselors, Definitive drug testing

## Abstract

We conducted a psychotherapeutic examination of the use of definitive drug testing (liquid chromatography with tandem mass spectrometry) in the treatment of substance use disorders (SUD). Employing a generic qualitative method (Caelli et al. in *International Journal of Qualitative Methods, 2*(2), 2003; Merriam, 2009) we asked SUD counselors to provide narratives about cases where drug testing had revealed new or unexpected information about clients’ drug-taking behaviors. Semi-structured interviews with 12 SUD counselors were conducted by phone and analyzed for themes derived from the literature. These counselors reported many new positive drug tests in clients previously believed to be adherent with treatment. Key themes assessed in counselors’ narratives included initial client denial that was often followed by later acknowledgement of relapse and increased motivation, at times presenting new opportunities for clients to engage in treatment and enhance the therapeutic alliance. These results suggest that definitive drug testing can be used in a non-stigmatizing and therapeutic manner.

Drug testing is an increasingly important component of the treatment of substance use disorders (SUD) in a variety of settings. For example, some of the commonly cited purposes of drug testing in the treatment of SUD are listed in the recent American Society of Addiction Medicine White Paper on drug testing (ASAM 2013). They include: verification of self-reports, confirmation of diagnoses, identification of denial and minimization of drug and alcohol use, enhancement of motivation for treatment, measuring biological adaptation, assisting in the development of treatment planning, monitoring of treatment response, documentation of treatment effectiveness and outcomes, supporting of client advocacy by validating abstinence from alcohol and drug use, and validation of adherence in taking prescribed controlled substances. In this view, linking drug testing with psychological variables associated with the treatment of SUD, such as denial (Blume and Marlatt 2009; Levin 1998) and motivation (Prochaska et al. 1992), suggests a potential psychotherapeutic role of drug testing.

This recent reconceptualization of drug testing is striking given its historical roots in a forensic model, such as in workplace and criminal justice settings (Substance Abuse and Mental Health Services Administration [SAMHSA], 2012), and as such may carry with it some stigma that should be considered in clinical and therapeutic contexts (Reisfield 2014; SAMHSA 2011). In contrast to the forensic use of drug testing with its focus on public safety, such as keeping intoxicated truck drivers off the road, the goal of clinical drug testing is on improving health outcomes of the patient (SAMHSA 2012). Moreover, the forensic model relies largely on immunoassay (IA) technology, which while sufficiently suited to its original purpose lacks the sensitivity and specificity of definitive, mass spectrometry drug testing. Mass spectrometry (e.g., gas chromatography mass spectrometry [GC-MS] or liquid chromatography tandem mass spectrometry [LC-MS/MS]) was traditionally used to confirm positive results of IA tests in a sort of two-step process, which is why it has been historically referred to as “confirmatory testing.” However, when medically necessary, drug testing can be performed directly with mass spectrometry, bypassing the limitations of the IA step. Definitive drug testing is more specific and sensitive than IA and is prone to much fewer clinically false negatives and positives (Pesce et al. 2010; Reisfield et al. 2009). Definitive drug testing may be an important tool in clinical settings and in the treatment of SUD (ASAM 2013; Gourlay et al. 2012; Owen et al. 2012).

Potential obstacles to drug testing have been acknowledged in the literature, including concerns of stigmatizing clients, burdening the therapeutic relationship, or possible legal consequences for clients (Reisfield 2014; Gourlay et al. 2012; SAMHSA 2011). As SUD are already prone to many sources of stigma (Earnshaw et al. 2013), any investigation of the potential therapeutic impact of drug testing should include consideration of potential stigma and other obstacles. Few studies have examined the clinical impact of drug testing, (Clancy et al. 2013; Reisfield et al. 2007), and to our knowledge none have specifically investigated the potential psychotherapeutic role of drug testing in the treatment of SUD. Thus, the focus of this paper is to address this need through a generic-qualitative (Caelli et al. 2003; Merriam 2009) examination of how drug testing may impact treatment at the individual level, from the point of view of counselors’ work with their clients. The purpose of this study has two components: The first is to provide some insights from SUD counselors, through our own psychotherapeutic lens, to other counselors who are, or are considering, integrating clinical drug testing into their work with clients. The second purpose is to provide preliminary data to help generate hypotheses for future studies as part of a progression toward further developing this line of inquiry.

It should be noted that, in many states, counselors are not authorized to independently order clinical laboratory tests (including drug testing). But, members of a clinical team can use a baseline understanding of drug testing to help understand this objective data about their clients. Learning how to understand drug test results may create an opportunity for use in therapy efforts with clients/patients.

## Current Study

In order to help develop a greater understanding of how drug testing may impact the treatment of SUD, we sought detailed narratives and perspectives from substance-use counselors about the clinical use of definitive drug testing (LC-MS/MS) and its potential impact on their work with their clients. The narrative data presented here is part of a larger study that included treatment plan, patient-outcome, and drug-testing knowledge questions. A critical goal of the present study was to let the narratives unfold, potentially to discover the unexpected. An important and common feature among these vignettes was that they represented a transitional period in this cohort when a facility-wide change was implemented, switching drug testing methods for all clients from a laboratory employing the forensic model in which only IA-positives were confirmed by mass spectrometry, to a laboratory utilizing a definitive model, in which all specimens were processed only through LC-MS/MS. Given the power of the newer testing methodology, a greater frequency of actual positive test results in previously “negative” individuals was anticipated. However, what was unknown was how clients and their counselors would respond, or the potential impact on the therapeutic community as a whole. An unanticipated response was tension in the community during the transition, the likely reaction to many unexpected positive results discovered in clients often mandated and previously thought to be abstinent. Indeed, as one counselor stated,

We are getting a lot more positive results right now which everybody is sort of reeling from. It’s been very disruptive, it’s difficult, but I think it’s really important because otherwise [how do we] know what are we actually doing? So I definitely think it’s a pro. You know the con is obviously the learning curve because we are switching from something that was sort of permissive so now everyone has to adjust.

There seemed to be a growing realization that the community might have to reevaluate their culture and how they deal with denial and that there was much work remaining in order to help clients achieve their goals of sobriety. In fact, part of the impetus for this study was the spontaneous and passionate revelations from many counselors about how their understanding of their clients, and their work with them, had changed in the face of the new drug testing that provided more specific clinical data. They wanted to get their stories out and share them with the rest of the SUD-treatment world.

## Methods

In this IRB-approved study, SUD counselors volunteered at two large urban substance-use treatment facilities, one residential and the other an outpatient program, where all clients were concomitantly human immunodeficiency virus (HIV) positive and had recently been started on, or switched to, laboratory-definitive LC-MS/MS drug testing from a laboratory that employed the more traditional forensic model in which only IA positives were confirmed by mass spectrometry. This study occurred over a period of approximately 2 months and started approximately 3 months after the entire facility had switched drug testing procedures. Additionally, when these treatment facilities made the switch to LC-MS/MS, they also chose to rely almost entirely on oral fluid testing for this purpose. Oral-fluid drug testing can provide the advantages of easier observed collections and to pose a potentially greater obstacle to adulteration (Conerman et al 2014; Heltsley et al 2012).

### Participants

All counselors at these two settings were offered the opportunity to participate, and nearly all did. (We say “nearly” because it remained unclear whether there had been a small amount of staff turnover during the course of this study.) The inclusion criteria were that counselors had worked directly with clients in a therapeutic capacity regarding their SUD and that the work had included discussion of drug-test results. We considered this sampling method to be adequate to address the purpose of the study (Merriam 2009). Each counselor was initially asked to think of two cases in which the newly implemented definitive drug testing had an impact on treatment. After reviewing and signing informed consent, counselors were contacted by phone, at the time and number of their preference, by a trained research assistant utilizing a semi-structured interview and instructed to not reveal any identifying information about their clients. With the counselors’ permission, interviews were recorded and transcribed. Counselors were told that the interview would take approximately 30 min and that they would be compensated $50 each for their time.

#### Residential Facility

The majority of clients in the residential facility were mandated by the criminal justice system for substance treatment, typically for 12 to 18 months. All clients had a history of SUD and were HIV positive. Many entered the program straight from a detoxification program (“detox”). Frequency of testing included a baseline drug test for every client, but thereafter as medically indicated (e.g., based on risk factors such as history and aberrant behaviors). After completion of the program, many clients had the opportunity to remain at the facility, and some had been there approximately 2 years. The residential facility referred to itself as a modified therapeutic community within a nursing home for people with AIDS, employing a number of modalities, including individual and group therapy sessions scheduled throughout the day. The intensity of treatment, such as the number of groups assigned to a given client, was based on individualized assessments. A quasi-harm-reduction model was employed wherever possible as long as clients were making progress toward the goal of sustained abstinence. As clients progressed through the program, they acquired more independence and privileges, including the ability to leave the residence under certain conditions. Clients who had adherence issues could be assigned more frequent therapy sessions, additional education, closer monitoring, and potentially decreased privileges such as temporary suspension of weekend passes. Drug tests were ordered and interpreted by physicians as medically indicated, and the results were shared with staff and clients wherever appropriate.

#### Outpatient Facility

As with the residential program, many clients were court mandated to treatment in this Adult Day Treatment Program and all were HIV positive and had a history of SUD. Some clients were admitted directly from the residential program, and many of the attributes of this population were the same as for the residential program described above. Level of participation varied, whether weekly, daily, or part of a day. There was a variety of substance-related and health education groups and individual therapy. Drug testing occurred randomly and as medically indicated, which produced an average frequency of approximately once per week.

### Materials

A semi-structured interview was designed by three PhD-level psychologists (first, sixth, and last authors) experienced in substance-use disorders and drug testing (see Appendix for the full interview form). Some interview items were open-ended to illicit narratives about clinical cases (Merriam 2009), and some were drawn from the literature (ASAM 2013; Clancy et al 2013; Gourlay et al. 2012; Reisfield et al 2007). The main scripted prompt that initiated the counselors’ narratives was, “Without revealing any identifying information, please tell a story in which a client was recently started on or switched to the new LC-MS/MS laboratory drug testing and where that change had an impact.” This open-ended item was designed to illicit the sorts of narratives that counselors had spontaneously reported to the first and last authors during educational programs related to drug testing. The educational programs are part of the usual services made available by the drug-testing laboratory and were requested by the medical director of the treatment center (second author) in response to many new questions from counselors about the testing. An experienced research assistant was trained by the principle investigator on conducting the interview by phone. Mock interviews were repeated until the assistant demonstrated an adequate level of consistency.

### Procedures

Substance-use counselors volunteered via sign-up sheets and signed informed consent forms available at staff meetings at the two treatment locations. Counselors were interviewed via telephone and informed that the interview would take approximately 30 min to complete. Counselor interviews were recorded, with permission (all counselors consented to recording), using an *ESONIC Mobile Phone Call Recorder & Voice Recorder*. Following demographic questions, the core of the interview consisted of the open-ended prompt for narratives, followed by optional clarifying prompts, and finally specific items related to treatment-plan effects and client outcomes (not reported here).

### Analytic Approach

The interviews were transcribed by a research assistant and cleaned of any potential identifying information. In addition to tabulating responses to discrete interview questions, transcripts were coded for thematic content by two PhD-level clinical psychologists (first and last authors) with expertise in substance-use and drug testing. A priori guidance for thematic content was provided by ASAM (2013), and in particular the potentially psychotherapeutic themes of denial (Blume and Marlatt 2009; Levin 1998) and motivation (Prochaska et al. 1992) were of interest. Thematic a priori guidance was also provided by the initial spontaneous reports from counselors, which, according to our clinical-psychologist perspective, spoke to potential therapeutic relationship effects (Kelley et al. 2014; Levin 1998). These three a priori themes were also built into the clarifying prompts of the semi-structured interview, increasing the likelihood that they would be represented in the data. The fourth theme of potential stigma was not built into the interview but was suggested by the literature (Earnshaw et al. 2013; Reisfield 2014; SAMHSA 2011) and emerged from initial reviews of the narratives, as agreed by the two reviewing psychologists. The coding by the two reviewing psychologists based on these four themes was in complete agreement. The first author selected excerpts based on this coding, which were then approved as being consistent with and representative of the coding by the other two psychologist authors. Coding was not mutually exclusive, i.e., a given excerpt could represent multiple codes. The coded themes provided organizational and conceptual structure to the presentation of the data but were not meant to represent all possible themes contained in the narratives (Merriam 2009). The number of times each coded them occurred was not counted, as the relative frequency of each of these themes was not considered to be interpretable or meaningful.

To enhance scientific rigor (Caelli et al. 2003; Merriam 2009) and help ensure the clinical relevance and representativeness of the selected data, a subset of excerpts (judged as representative by the first author) were presented to a group of 10 of the original participant counselors and the medical director for feedback on whether the excerpts and associated themes had captured what they felt was important and useful for the purpose of this study. The group represented merely those that were available to attend on a date of convenience. Confirmation from this group was obtained verbally and noted by the first author. While the feedback process did spontaneously illicit some additional minor detail, none was deemed to substantively alter the data.

As this is one of the first studies of its type, the data was treated as descriptive and exploratory; inferential statistics were not utilized. Demographics are represented as frequencies, means, and percentages. A secondary analysis of client cognitive change was based on the temporal stability of client denial. This is useful because the ways that individuals may move in and out of denial is relevant to better understanding factors that could influence therapeutic relationships (Levin 1998). Only descriptive results were provided for this measure, as the sample size would not permit calculation of meaningful correlations.

## Results

Counselors (*n* = 12) were split evenly among the two settings, and their demographics were comparable with the exception that counselors were, on average, older in the residential compared with outpatient settings, with an average age of 48.7 and 36.6 years, and an average number of months working with clients of 136.5 and 71.5 months, respectively. The complete sample of counselors was 75 % female, 25 % African American, 25 % Caucasian, 16.7 % Latino, and one third “other.” Eight had master’s degrees, three had bachelor’s degrees, and one had not completed high school. Four counselors were social workers, one was a psychiatric nurse practitioner, and the rest described themselves as counselors or case workers. All participants described their specialty as related to substance-use, worked directly with clients as counselors, work that typically included discussion of drug-test results. Counselors provided vignettes encompassing 24 unique clients, with an average age of 51.4 years; 37.5 % were female, and 75 % were court-mandated to treatment. The average duration of SUD was ~28 years (range 10–45), with the self-reported primary drug of choice being 41.7 % crack cocaine, 33 % heroin, 20.8 % powdered cocaine, and 4.2 % prescription-type drugs. Most clients had histories of using multiple illicit substances.

### Narrative Data

An important element common to most of the vignettes was of newly discovered relapses or continued use being identified with the switch from the IA based laboratory to the LC-MS/MS laboratory. This was not unexpected given the greater sensitivity and specificity of LC-MS/MS testing, and that counselors were prompted to think of cases where the change in drug testing was associated with impact on treatment. Interestingly, however, clients had, in some cases, apparently become accustomed to the previously employed IA model of testing, expecting their continued use to go undetected. Importantly, initial denial of test results was common and at times energetic, perhaps owing in part to legal consequences or stigma. As one counselor illustrated,

I see a lot of the residents, especially this particular young lady, she figured that if she knew her test was Monday and she did stuff on the weekend that nothing would show.

This counselor described the client’s first encounter with the newly implemented LC-MS/MS testing, reporting:

…I sat her down, I had the results in my hand, and I asked her how she was doing, kinda ease in to it. Asked her where did you go? How are things going? Because your tox came back positive for whatever. She got very upset very agitated. Walked out the office was cussing me out saying I was lying…

However, in many cases the initial denial subsided as counselors developed confidence in their understanding of the testing methodology and relayed education about the improved accuracy and sensitivity of the tests that they had received from educational services provided by the newly employed lab. As one counselor explained, definitive drug testing could provide an opportunity to:

…foster healthy conversations with clients about their drug use. You can kind of catch people when they are not telling the truth or when they are in denial. So if someone is in denial you can kind of work on helping them in another way.

The change in testing methodologies was not always clinically impactful. For example, in one case in which the client was known for a long history of dispositional intransigence, neither the newer testing nor enhanced therapeutic and educational efforts seemed to lead to any meaningful changes, with an incarceration being the final result at the time of this study. Nevertheless, in most cases, clients who had started testing positive, often for cocaine and alcohol, quickly began testing negative, suggesting increasing abstinence as they came to accept the validity of the newer testing methodology. Many of the counselors indicated a favorable view of this change, as they came to realize that they were seeing their clients’ substance-use behaviors more clearly.

Counselors also spoke of how newly revealed continued use at times impacted the therapeutic community. One counselor told a story of a popular female client who had been viewed by the community as a “model of sobriety” because she had consistently tested negative prior to implementation of the LC-MS/MS definitive laboratory. The counselor explained being surprised that:

…she was not ready to stop using …we were able to pick up on that after we caught her relapses …she was probably doing it before but because of the [previous IA-lab] testing we were unable to pick it up. Whatever games she played before it didn’t work [any more].

Initially her denials were dramatic but eventually she entered what may have been one of her first true periods of abstinence, as suggested by consistently negative LC-MS/MS test results. Nevertheless, during a weekend pass she died tragically following a relapse associated with marital discord. Importantly, the counselor explained,

Her peers look at her and see her and see that they might have learned a lesson. They think ‘wow, I thought she was doing good (sic) but she continued to relapse.’ …knowing that she was continuing to use …they probably learned a lesson because there is a saying in the room that some of us will die for others to live…

Initially, pushback from the community created stress among the staff, though most began to see it as an opportunity to help improve outcomes with clients. In many cases, counselors indicated that they recognized a new level of motivation among their clients, sometimes accompanied by a deeper commitment to the program and to therapy.

In some cases, initial denial that seemed to be related to potential legal consequences turned out to belie more personal and emotional layers that eventually may have led to enhancing the therapeutic relationship. One counselor recalled working with a mandated 50-year-old female client who was being treated for a history of crack cocaine use. IA tests performed by the criminal justice system had been coming up negative weekly, but the treatment center found cocaine positive with LC-MS/MS (likely a result of a large difference in sensitivity between the two testing methods). Initially she denied use, possibly encouraged by the negative test results coming from criminal-justice, and also potentially related to her fear of legal consequences. But ultimately the center was able to continue working with her, and her denial gave way to greater honesty and making further progress with her recovery. As the counselor stated, the positive cocaine result with LC-MS/MS was:

…a catalyst therapeutically as far as acknowledging that there was a recent relapse and figuring out what we now had to do to move forward. …I think that she maybe opened up a little bit more about guilt and disappointment in herself. I think there was a vulnerability that happened but it was therapeutically useful.

As a result of the relapse, the criminal justice system added 2 months to her treatment mandate but she did not return to prison. She was given more structure and monitored more closely. The counselor indicated she became more engaged in treatment. By the time of this data collection, she had remained abstinent for several months, as indicated by LC-MS/MS testing, and was on her way to successfully completing the program.

Hidden emotional components were not always accompanied by denial. One counselor described a 56 year-old female with long history of polysubstance since her 20s. Upon switching from the IA lab to the LC-MS/MS lab, continued use of clonazepam was revealed after more than a year of treatment and consistently negative IA toxicology. Upon her first positive LC-MS/MS test for clonazepam (a common benzodiazepine) there was no denial. Rather,

…there was a deep sense of shame and failure …she was tearful and crying and saying the moment that she [relapsed] she wanted to go straight to her staff counselor and tell him but she couldn’t bring herself to disappoint him in that way. …it was kind of like she was waiting for [the positive LC-MS/MS result] to happen so she could say it.

She entered a new level of progress in her therapy, revealing old sources of emotional pain she related to SUD. She was monitored more closely and tested negative for several months. She recently experienced a new relapse, but remarkably, this time, she did not wait for the positive toxicology to report it. Her previous experience had helped to build a new level of confidence and trust in the support from her therapist and others in the community. As a result, this new relapse was much shorter and less intense.

As SUD may be best understood as psychobiological problems involving interplay of the biological aspects of the drug with the personality and interpersonal world of the client, psychotherapy focusing on substance use will often include interpersonal and relational work, helping develop the client’s sense of self in the process (Levin 1998). Accordingly, one counselor explained how working through the denial of a positive drug test in therapy paralleled an emerging sense of authenticity that may have extended to new relationships. The counselor recalled a mandated, 47-year-old male client with a decades-long history of polysubstance use. He had, for the first several months in the program, produced “clean” or negative urine drug tests with the traditional IA-lab testing. He was considered a model community member until his first drug test with the newly introduced LC-MS/MS that produced an unexpected positive result for cocaine. In keeping with the program’s policy, he was consequently put on a 30-day privilege restriction and required to attend more daily groups, and subject to ongoing drug monitoring. As his counselor stated,

It was positive for cocaine and this was totally unexpected as he was one of those model citizens of the program, he did everything and always supportive of his peers. …he denied it…

But as the counselor explained,

One of his big issues was loneliness and not being able to connect authentically with people…” His counselor stated that this lack of interpersonal authenticity “paralleled” his inauthentic sobriety. “Then we kind of took a switch… he actually started expressing what I believe were authentic feelings of being supported, being cared for…

The client slowly started to speak more openly in therapy about his continued substance use, and during this time he began being more open with a romantic interest, at first uncharacteristically revealing his HIV status and eventually his substance use problems:

…he admitted that he had a substance use problem and that he’s been in treatment, and this cascade of disclosures and honesty came post tox (after the positive cocaine test) and I feel like that there is a direct link…

After maintaining a period of actual sobriety, suggested by the LC-MS/MS tests, the client was preparing for a successful discharge from the program and planning to move in with a woman with whom he had been honest about his history.

One theme that emerged was of the utility of monitoring more closely during certain periods of heightened vulnerability, such as during the common transition from a more highly structured environment to less. For example, one counselor recalled working with a male client in his 60s, with a 20-year history of incarceration and cocaine use who had recently been mandated to an outpatient treatment setting after graduating from residential programming where he had come directly from prison. He had tested negative for months until LC-MS/MS was implemented, and then tested positive for cocaine. Education and intensified treatment were provided. Legal consequences remained a possibility. Despite maintaining his initial denials, he became abstinent or substantially decreased his use (as suggested by consistently negative LC-MS/MS tests), which may have provided him greater opportunity to become more invested in therapy. As the counselor recalled,

Now, the conversations [are] becoming not only about treatment but he’s opening up and letting me enter his personal life. He is talking about relationships he has with his children which he never really did before…

From this counselor’s perspective, certain clients like this need some additional monitoring during what may be high-risk transitional periods where they are slowly being reintroduced to greater personal responsibility after a period of having their lives and the choices controlled by others:

Once a person comes out of inpatient program and released back into the community I think it’s sort of a shock to them. More of a shock and excitement that they are out into the community and they are able to leave the program and go home to their apartment like a regular person where as in an inpatient they are confined and it’s a controlled environment.

### Client Cognitive Change

In order to approximate clients’ cognitive change we assessed initial denial of unexpected definitive drug-test results, including denial of relapse or continued use, and whether they later acknowledged it. There were 12 (50 %) clients who had initially denied unexpected results and then later admitted to relapse or continued use. Nine clients (38 %) continued denial throughout treatment despite positive definitive test results, and the remaining 3 (13 %) initially acknowledged the accuracy of the results. This brief analysis supports the notion that denial is not static but rather has temporal variability.

## Discussion

To our knowledge, this is the first study of counselors’ perspectives of the impact of definitive drug testing in the treatment of SUD. Counselors reported their experiences utilizing the results of drug tests with their clients at a time when these treatment centers had recently switched from an IA-based, traditional forensic model of IA based drug testing to the more sensitive and specific definitive LC-MS/MS drug testing via oral fluid. These counselor’s narratives provided an opportunity to explore how clinical drug testing in SUD treatment can impact denial, motivation, the therapeutic relationship, stigma, and some of the interrelations among these. More broadly, these counselors indicated that the switch to definitive drug testing provided them, in many instances, with new insights into their clients’ actual drug-use behaviors and opportunities to work with them in new ways to better help them achieve their therapeutic goals.

Identifying denial and enhancing motivation are among several potential benefits of clinical drug testing proposed by the American Society of Addiction Medicine White Paper on Drug Testing (ASAM 2013). These data suggest that working with denial and incorporating the results of definitive drug testing into treatment in a non-stigmatizing manner may, in some cases, provide the conditions for greater engagement in therapy and enhancing the therapeutic relationship (Kelley et al. 2014). This last point is particularly noteworthy given that there has been speculation that drug-test results in the clinical setting might unintentionally stigmatize clients and damage the therapeutic alliance (e.g., Reisfield 2014; SAMHSA 2011). Stigma in the context of SUD can often represent the negative attitudes of healthcare professionals that have clinical consequences for patients (Earnshaw et al. 2013). In these counselors’ narratives, we mainly found anticipated stigma in the form of embarrassment or shame on the part of the clients, as reported by counselors. We were encouraged, however, that in the cases where such feelings were revealed, counselors found it to be an opportunity to deepen their work with clients. Although these data are necessarily preliminary, they clearly point to the potential for counselors and their clients to overcome potential stigma and other obstacles in order to better utilize the objective information provided by definitive drug testing. Of note there were descriptions in some narratives of how catching relapses and denial created an opportunity to work with clients in new ways, such as fostering constructive dialogues, consistent with a number of guidelines related to the use of clinical drug testing in both substance use and chronic pain (ASAM 2013; National Institutes of Health [NIH], 2009; Gourlay et al. 2012).

As captured in counselors’ narratives and supported by the brief analysis of client-cognitive change, denial (Blume and Marlatt 2009; Levin 1998) appears to have temporal variability, suggesting opportunities and targets for change in the course of treatment. Other aspects of denial should be considered as well. For example, clients might have outwardly denied continued use revealed by definitive drug testing, despite being aware of the truth. This might be consistent with a fear of potential legal consequences. Alternatively, some cases of denial may have involved self-deception, or maintaining a lack of awareness of the true extent of their own drug-use behaviors. In this sense, shifts in denial may, at times, be associated with changes in awareness. By extension, shifts in denial might also be related to motivation, in that progressing through levels of readiness to change may be related to shifts in awareness and opportunities to observe oneself engaging in new behaviors (Prochaska et al. 1992). Such would be consistent with the perspective that the enhanced accuracy of the newly introduced definitive testing may have led to new periods of abstinence or reduced use, as suggested by counselors’ reports of instances in which unexpected positive test results were followed by negative LC-MS/MS drug tests. But perhaps most interestingly, in some instances such changes precipitated an increase in clients’ engagement in therapy, suggested at times by a greater openness to discuss guilt or shame related to substance use and emotions around damaged relationships (Levin 1998). In either case, it seems reasonable that any time spent in genuine abstinence likely increases the prognosis of a better outcome over time.

A less expected but potentially important theme emerging from these counselors’ stories was that individual differences in clients’ traits and temporal circumstances seemed to impact the clinical effects of drug testing at times. For example, in a few cases in which the clients seemed to have more of an antisocial disposition or a lower readiness to change, changes in drug-use behaviors may have been relatively superficial, appearing more connected to the immediate goal of completing the program (Prochaska et al. 1992). However, particularly instructive was the perspective of one counselor who identified the use of clinical drug testing as key during a potentially sensitive period of relapse risk when clients are transitioning from highly structured and controlled environments to ones with greater autonomy. As this is not an unusual circumstance, it may represent an opportunity for counselors to better identify changing patterns of risk factors in their clients that could be addressed with enhanced monitoring and treatment. An important area of future inquiry will be to examine a broader range of traits and situations that impact the therapeutic effects of clinical drug testing.

Following the initial stress of adapting to the changes in testing methodology, identifying relapses, enhancing motivation, and deepening the therapeutic relationship may have contributed to a more helpful environment for the therapeutic community as a whole. Counselors spoke at times about how a culture develops among clients in the community in which they share their stories, advise each other, and develop a shared perception of treatment and their role in it. In listening to these counselors, there was a sense at times of a cultural shift in the client community from an attitude of “let’s game the system” to one of “how can we adapt to actually being sober.” This is consistent with counselors’ reports that the initial community unrest that coincided with the introduction of the definitive drug testing eventually began to soften. To the extent that there may have been a genuine evolution in the attitude or culture of the community, it could have a desirable impact on the recovery efforts of individual clients. This is a compelling notion that will, for now, remain the work of future research to explore.

### Limitations and Future Directions

As the results from this qualitative study are exploratory and descriptive, caution is warranted in interpreting them. The present findings provide some insights for counselors doing similar work, and preliminarily support for the relevance of denial, motivation, the therapeutic relationship, and stigma in better understanding the role of clinical drug testing in the treatment of SUD. The present findings also suggest that quantitative studies may be warranted to examine, for example, the extent to which these effects lead to changes in treatment-plans and client outcomes. The limited sample, while appropriate for the goals of the present study, limits the generalizability of the findings, such as in non-urban settings or populations without mandated clients or who are not HIV positive. Nevertheless, this first examination of counselors’ perspectives of the use of definitive drug testing in their work with substance-use clients is consistent with that proposed by ASAM (2013) and others, warranting further investigation.

## Conclusions

Counselors’ perspectives of the impact of definitive LC-MS/MS drug testing were consistent with potential benefits outlined in a number of guidelines (e.g., ASAM 2013; NIH 2009; Gourlay et al. 2012) including the treatment goals of increasing motivation (Prochaska et al. 1992), reducing denial (Blume and Marlatt 2009; Levin 1998), and, by extension, enhancing the therapeutic relationship (Kelley et al. 2014; Levin 1998). They further suggest that counselors and their clients can overcome stigma and other potential obstacles to the therapeutic use of definitive drug testing (Reisflield 2014). Though these qualitative results are necessarily preliminary and must be interpreted with caution, they are consistent with previous work suggesting that clinical drug testing can impact treatment decisions and patient outcomes to help optimize patient care (Clancy et al. 2013; SAMHSA 2011). The current results extend previous research by illustrating and elaborating the impact of definitive drug testing, as a specific case of clinical drug testing, in the treatment of substance use disorders. Substance-use counselors may be able to utilize definitive drug testing in the early identification of relapses and then work with potential denial and stigma, enhancing motivation and the therapeutic relationship.

‘All procedures followed were in accordance with the ethical standards of the responsible committee on human experimentation (institutional and national) and with the Helsinki Declaration of 1975, as revised in 2000 (5). Informed consent was obtained from all patients for being included in the study.’
